# Recurrent pancreatitis caused by previous coiling of a pancreaticoduodenal artery aneurysm in a patient with median arcuate ligament syndrome

**DOI:** 10.1055/a-2113-9880

**Published:** 2023-07-27

**Authors:** Haruka Hagiwara, Ko Tomishima, Yasuhisa Jimbo, Mako Ushio, Akinori Suzuki, Toshio Fujisawa, Hiroyuki Isayama

**Affiliations:** Department of Gastroenterology, Graduate School of Medicine, Juntendo University, Tokyo, Japan

Median arcuate ligament syndrome (MALS), reported by Harjola in 1963 1 , involves a celiac artery stricture caused by compression of the ligament, resulting in reduction of blood perfusion to the abdominal organs. Rupture of a pancreaticoduodenal artery aneurysm has a high mortality rate of 14.3 %, even when treated with emergent transcatheter coil embolization 2 . We report a case of recurrent pancreatitis caused by main pancreatic duct (MPD) obstruction by a perforated coil after transcatheter embolization of a pancreaticoduodenal artery aneurysm caused by MALS.

The patient was a 53-year-old man who had undergone prophylactic coil embolization 2 years previously for a 6-cm inferior pancreaticoduodenal artery aneurysm. Contrast-enhanced computed tomography revealed celiac artery stenosis due to the median arch ligament ( Fig. 1 ), and he underwent median arcuate ligament release to prevent recurrence. However, 3 months after coil embolization, the patient had repeated episodes of pancreatitis, and obstructive pancreatitis due to pancreatic duct stenosis at the coil embolization site was observed. Endoscopic retrograde cholangiopancreatography (ERCP) was performed and outflow of murky fluid from the papilla was observed ( Fig. 2 a ), and the contrast medium simultaneously penetrated from the body to the tail of the pancreatic duct, the bile duct, and the aneurysm cavity ( Fig. 2 b ). Transpapillary drainage was attempted, but the catheter could not be passed beyond the stenosis. Peroral pancreatoscopy (POPS) revealed the metal coil, which was exposed in the MPD ( Fig. 3 ; Video 1 ). The MPD obstruction was considered to have been caused by the perforated coil. The coil was removed endoscopically but this failed to resolve the MPD obstruction. Endosonography (EUS)-guided pancreatic duct drainage was performed to place a plastic stent into the duodenum via the Santorini duct 3 . The patient’s recurrent pancreatitis was finally resolved after EUS-guided pancreaticogastrostomy with antegrade stenting to bridge the accessory papilla.

**Fig. 1:**
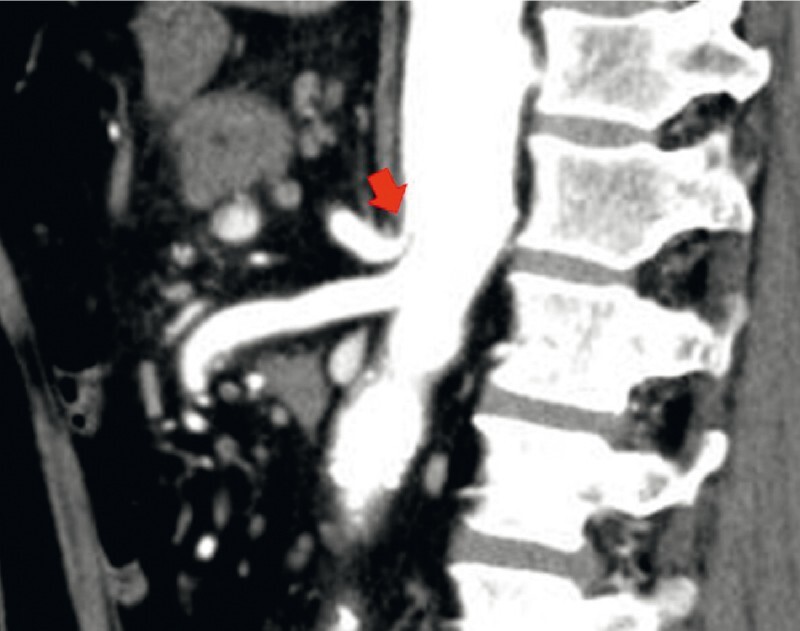
Contrast-enhanced computed tomography scan image showing celiac artery stenosis due to the median arch ligament (arrow).

**Fig. 2:**
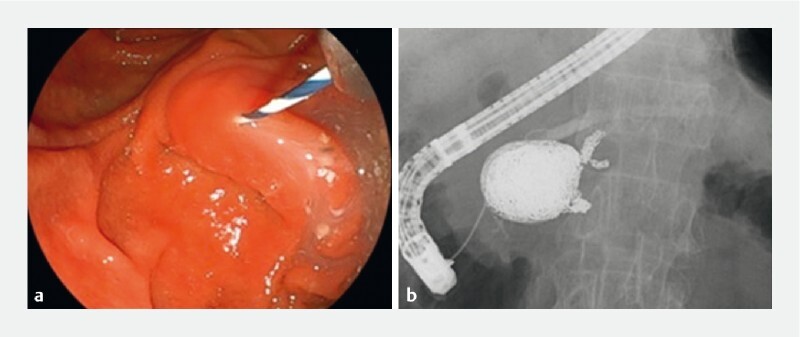
Images during endoscopic retrograde cholangiopancreatography showing: **a** murky pancreatic juice oozing out from the pancreatic duct; **b** contrast medium simultaneously passing from the body into the tail of the pancreatic duct and the aneurysm cavity.

**Fig. 3:**
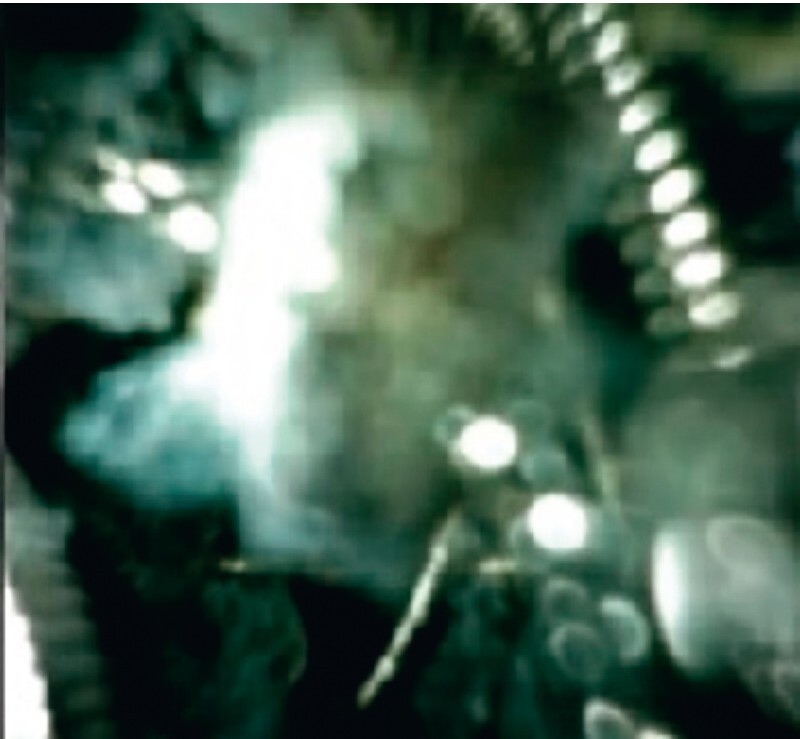
View during peroral pancreatoscopy showing the exposed metal coil within the main pancreatic duct.

**Video 1** The exposed coil is seen in the main pancreatic duct (MPD), which was causing obstructive pancreatitis. The coil is successfully removed from the MPD, but it was not possible to perform transpapillary drainage. Endoscopic ultrasound-guided pancreaticogastrostomy is therefore performed.


Endoscopy_UCTN_Code_TTT_1AS_2AD

## Notes

Endoscopy E-Videos

https://eref.thieme.de/e-videos
*E-Videos* is an open access online section of the journal *Endoscopy* , reporting on interesting cases and new techniques in gastroenterological endoscopy. All papers include a high-quality video and are published with a Creative Commons CC-BY license. Endoscopy E-Videos qualify for HINARI discounts and waivers and eligibility is automatically checked during the submission process. We grant 100% waivers to articles whose corresponding authors are based in Group A countries and 50% waivers to those who are based in Group B countries as classified by Research4Life (see: https://www.research4life.org/access/eligibility/ ). This section has its own submission website at https://mc.manuscriptcentral.com/e-videos
